# *Aspergillus terreus* Meningitis in Immunocompetent Patient: A Case Report

**DOI:** 10.3389/fmicb.2015.01353

**Published:** 2015-12-01

**Authors:** Abdelrahman Elsawy, Hani Faidah, Abdalla Ahmed, Asmaa Mostafa, Farah Mohamed

**Affiliations:** ^1^Medical Microbiology Department, Al-Noor Specialist Hospital, Ministry of HealthMakkah, Saudi Arabia; ^2^Department of Microbiology, Al-Azhar Faculty of Medicine, Al-Azhar UniversityCairo, Egypt; ^3^Department of Microbiology, College of Medicine, Umm Al-Qura UniversityMakkah, Saudi Arabia; ^4^Department of Microbiology, Tanta Faculty of Medicine, Tanta UniversityTanta, Egypt; ^5^Department of Medicine, Al-Noor Specialist Hospital, Ministry of HealthMakkah, Saudi Arabia

**Keywords:** Aspergillus, terreus, meningitis, immunocompetent, patient

## Abstract

We present a description of a rare but dangerous case of fungal meningitis caused by *Aspergillus terreus* in an immunocompetent patient with a history of sinus disease.

## Background

Central nervous system (CNS) infection is one of the most disabling and deadly diseases worldwide. According to the World Health Organization, there were about 700,000 cases of meningitis in 2004, with approximately 340,000 related deaths (Mathers et al., 2004). CNS infectious pathogens include, infection with bacteria, viruses, fungi, and parasites. Fungal infection is generally opportunistic and has gradually increased in recent years, mainly due to the increase in acquired immunodeficiency syndrome (AIDS), transplant surgery and drug resistance to antifungal medication. Cryptococcus neoformans infection is still the most common CNS fungal infection, whereas Aspergillus and Mucor are relatively uncommon causes of CNS infection (Qi, 2014). Aspergillosis CNS fungal infection may present with various features depending on the pathogenicity of a specific pathogenic species and the status of the host's immune response (Rallis et al., 2014). The most common primary sites of aspergillosis are lungs, paranasal sinuses, and ear canal (Baddley et al., 2003). Aspergillosis of the paranasal sinuses is infrequent and usually involves the species *Aspergillus fumigatus* or *Aspergillus flavus*. The maxillary sinus is the most commonly affected sinus (Akhaddar et al., 2008). Fungal rhinosinusitis (FRS) is divided into invasive and noninvasive diseases based on histopathological evidence of tissue invasion by fungi. Invasive FRS is classified as acute, chronic, or granulomatous. The non-invasive forms of fungal sinusitis are allergic FRS and the fungus ball (fungal mycetoma) (Chakrabarti et al., 2009). Invasion of the CNS can occur through direct extension from the paranasal sinuses, hematogenous dissemination though pulmonary/cutaneous sources (Walsh et al., 2008) or by Iatrogenic inoculation of Aspergillus through spinal anesthesia, neurosurgery, or epidural steroid injections (Antinori et al., 2013). CNS aspergillus infections are mainly caused by *A. fumigatus, A. flavus* and rarely by *Aspergillus terreus, Aspergillus oryzae, Aspergillus granulosus*, or *Aspergillus candidus* (Antinori et al., 2013). Although *A. terreus* is an unusual cause of CNS invasion, its natural resistance to Amphotericin B is linked to a higher mortality rate (Schwartz et al., 2007).

## Case report

Sixteen-year-old male patient from Burkina Faso presented to emergency department of Al-Noor Specialist Hospital, which is a Joint Commission International (JCI) accredited, tertiary care hospital in the Holy city of Makkah, Saudi Arabia. The patient presented with a history of fever; and recurrent convulsions, followed by a decreased level of consciousness.

A week before the presentation the patient had nasal polypectomy and sinus surgery in a private hospital. Two days post-surgery the patient developed headache with high-grade fever that did not respond to antibiotics. Headache became progressively worse and the patient started experiencing episodes of fits and loss of consciousness.

Assessment of vital signs on presentation revealed a temperature of 37°C, pulse of 112 beats per minute, and blood pressure of 121/97 mmHg. The physical examination revealed no meningeal signs, pupils were normal and reactive, and there was normal muscles tone. Glasgow Coma Scale was 6 (E1 M4 V1), so the patient was intubated and mechanical ventilation was initiated.

Laboratory testing revealed a leukocyte count of 10,380 cells per cubic millimeter, with 76.5% polymorphonuclear cells. ESR was 42 mm/h and CRP 7.97 mg/dl. The comprehensive metabolic panel; including liver-function tests, was within normal limits (Table 1). Analysis of the cerebrospinal fluid revealed an elevated protein level of 120 mg/dl (reference range, 15–45), low glucose concentration of 38 mg/dl (reference range of 40–70 mg/dl). Gram stain of CSF showed no bacteria, but white blood cells were increased at 4200 white cells/μl; 90% polymorphonuclear cells.

**Table 1:** **Patient's parameters during admission days**.

**Parameter**	**1**	**2**	**3**	**4**	**5**	**6**	**7**
WBC'S (10^3^/ul)	10.38	11.10	11.18	6.52	6.76	12.13	9.12
Hb (g/dl)	10.3	10.8	11.4	9.6	8.4	9.5	11.3
Urea (mg/dl)	27	27	39	106	145	153	167
Creatinine (mg/dl)	0.99	0.99	1.6	6.1	7.7	7.96	7.8
Blood glucose (mg/dl)	100	102	233	477	547	243	–
ALT(IU/l)	20	21	22	624	455	584	–
AST (IU/l)	26	27	49	1127	363	571	–

**Figure 1:**
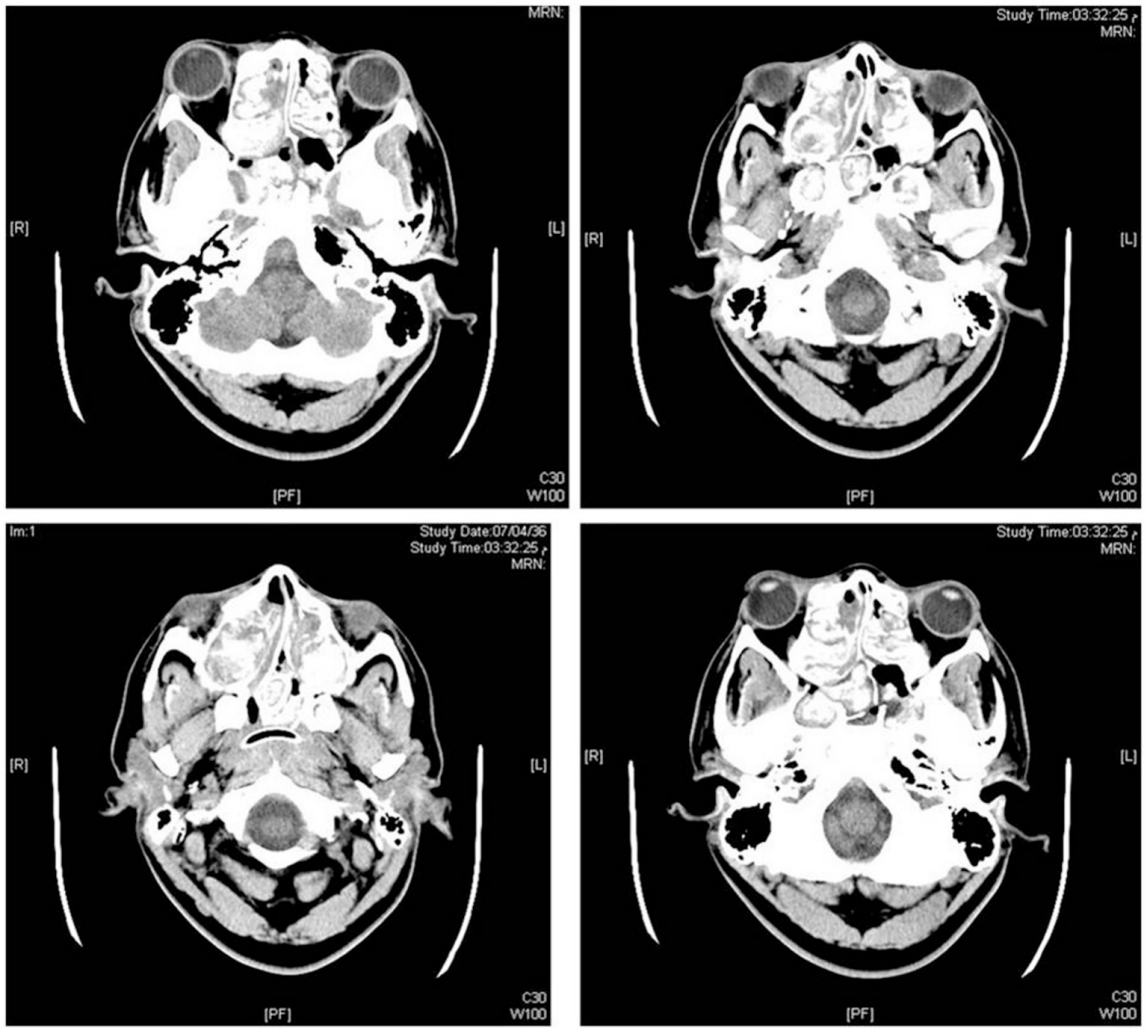
**Imaging studies**. Computed tomographic (CT) images of the head without administration of contrast, showing ethmoidal, sphenoid, and maxillary sinuses occupied by hyperdense soft tissue suggesting fungal sinusitis (bilateral sinupolyposis).

Computed tomography (CT) of the head showed dilatation of supra and infratentorial ventricular system with hydrocephalic changes. CT scan also suggested possible fungal sinusitis of ethmoid, sphenoid, and maxillary sinuses. Other findings were not remarkable and showed normal attenuation of cerebral cortex and white matter, no intra or extra-axial hemorrhage and normal posterior fossa structures (Figure 1). CT of the chest showed left lower lobe consolidation with right upper lobe linear atelectasis and mild ground glass density patches. Mediastinal vascular structures were normal with no lymph node enlargement, no pleural effusion, and normal chest wall.

The patient was started on intravenous vancomycin, ceftriaxone, acyclovir, metronidazole, clindamycin, and glucocorticoids.

Routine bacterial cultures of the blood and cerebrospinal fluid were negative, and microscopic examination of CSF revealed acute inflammatory cells (neutrophil leucocytes) and few admixed histocytes with conclusion of pyogenic meningitis.

On hospital day 3 the patient had signs of brain stem death in the form of dilated fixed unreactive pupils, loss of corneal and conjunctival reflexes and loss of gag reflexes. Brain death was confirmed twice clinically by neurology department with an interval of 24 h. Electroencephalography (EEG) was advised as proof of brain stem death by electrophysiological studies.

On hospital day 4, the patient showed abrupt elevation of blood glucose, urea, creatinine, and liver enzymes. EEG was done using brain death standard technique for 30 min and revealed severe cerebral dysfunction. Patient was continued on mechanical ventilation, but his blood pressure started to decrease. The patient immediately received Alpha/Beta agonists in an attempt to maintain his blood pressure at acceptable levels.

On hospital day 5, the microbiology laboratory reported a filamentous fungus growth in the CSF specimen, which was initially negative for bacteria. This positive CSF specimen was collected on the first day of admission. The filamentous fungus was later identified as *Aspergillus* species and the culture was sent to a reference mycology laboratory for species identification. Liposomal Amphotericin B and voriconazole were started on the same day, but patient continued to deteriorate. The patient died on hospital day 7 due to sudden cardiac arrest associated with severe meningioencephalitis, renal impairment, and septic shock.

### Isolation and identification of *Aspergillus terreus*

The CSF specimen was cultured in BACTEC FX blood culture bottles and incubated using BACTEC FX system. Three days after initial incubation, a positive culture signal was noted and fungal ball was noted inside the culture bottle. The fungal ball was directly inoculated onto blood, chocolate and Sabouraud's dextrose agar plates and incubated at 30°C. Filamentous fungal growth was obtained within 48 h and identification attempts were made by means of macroscopic and microscopic examination of the culture. Parallel CSF culture was performed according to standard procedure and all results were negative.

The CSF filamentous fungus isolate was identified as *Aspergillus* species by microscopic examination showing characteristic *Aspergillus* fruiting heads. For species determination Sabouraud's dextrose agar plate was sent to the Microbiology Research Laboratory, Department of Microbiology, College of Medicine, Umm Al-Qura University.

### Fungal its-based identification protocol

Washed fungal mycelium was used to isolate genomic DNA. A library for ITS metagenomics DNA sequencing was prepared using the Illumina Metagenomics Protocol and sequenced in Illumina MiSeq using version-2 500 cycles Nano kit. Species identification was determined using the Pairwise Sequence Alignment tool, (ISHAM ITS database http://its.mycologylab.org/). Sequence similarity equal to or greater than 97% was used as cutoff for species identification. Alignment of assembled ITS sequences showed high degree of similarity (above 99.7%) to *A. terreus* ITS reference sequences.

## Discussion

*Aspergillus* is a ubiquitous fungus in soil, water, decaying vegetation and organic debris, and was recognized to be pathogenic in humans as early as 1847. Abundant spore formation and septate dichotomously branching hyphae characterize the genus *Aspergillus*. Desiccation-resistant *Aspergillus* spores can be easily dispersed by air currents. Humans inhale hundreds of spores daily, which are usually handled by alveolar macrophages and neutrophils. There are more than 350 species of *Aspergillus;* however, only a few are pathogenic in humans. The most common pathogenic species are *A. fumigatus* and *A. flavus*, but many other species have been reported to cause infection in humans. Microscopic identification of *Aspergillus* is easy for some of the common species, but for many species, it might be difficult and need special expertise (Baddley et al., 2003).

The most common primary sites of aspergillosis are lungs, paranasal sinuses, and ear canal (Baddley et al., 2003). Sinusitis occurs in both healthy and immunocompromised patients. Healthy patients can present with signs and symptoms of chronic sinusitis or a mass (aspergilloma) in the maxillary or ethmoid sinuses. Immunocompromised patients present with invasive disease characterized by bony destruction with extension to contiguous sites such as the orbit or CNS (William Schwartz, 2012). FRS is often characterized as invasive or noninvasive diseases based on histopathological evidence of tissue invasion by fungi. Invasive diseases include (1) acute invasive (fulminant), (2) granulomatous invasive, and (3) chronic invasive. Noninvasive diseases include (1) saprophytic fungal infestation, (2) fungal ball, and (3) fungus related eosinophilia that includes allergic FRS (Chakrabarti et al., 2009). Although allergic FRS has a hypersensitivity etiology, a few reports document fungal invasion (Thakar et al., 2004).

Invasion of the CNS can occur through direct extension from the paranasal sinuses by hematogenous dissemination though a pulmonary or cutaneous source (Walsh et al., 2008), or by iatrogenic inoculation of Aspergillus through spinal anesthesia, neurosurgery, or epidural steroid injections (Antinori et al., 2013). Also, breach in the mucosal barrier, caused by inflammation or proliferation of fungal elements in the sinus lumen has been cited as a precipitating factor, due to imbalance in host-agent interactions and resulting in progression from non-invasive to invasive disease (Rowe-Jones, 1993).

Although *A. fumigatus* is the leading cause of invasive aspergillosis in immunocompromised individuals, *A. terreus, A. flavus, Aspergillus niger*, and rarely *Aspergillus nidulans* have been identified as etiological agents (Balajee, 2009). *A. terreus* is an emerging opportunistic pathogen causing fatal disseminated infections in immunocompromised populations with increasing incidence from < 2% in 1996 to 15% in 2001 (Baddley et al., 2003). Resistances of *A. terreus* to amphotericin B, thermotolerance and production of accessory conidia have been suggested as mechanisms that explain rapid dissemination of the organism during invasive infections (Blum et al., 2008).

The diagnosis of Aspergillus meningitis is challenging. In fact, diagnosis during life was obtained in only 55.9% of patients. Moreover, there is a reported higher frequency of diagnosis during life among immunocompetent patients (69.2%) as opposed to immunocompromised individuals (39%). This difference might be explained by a more aggressive and acute course of the disease in immunosuppressed hosts (Antinori et al., 2013).

The overwhelming bulk of literature on aspergillosis is concerned with immunocompromised hosts. Notable case series of craniocerebral aspergillosis occurring in apparently immunocompetent hosts have so far been reported mainly from India, Sudan, Pakistan, Saudi Arabia, UAE, and a few other African countries. On the contrary, reports from the West are largely confined to isolated case reports. The reason for this demographic difference is not clear, and a plausible explanation may be the hot, dry climate, and low socioeconomic status in the above-mentioned regions favor the growth of *Aspergillus* (Shamim et al., 2007; Antinori et al., 2013). Antinori et al. reviewed a number of Aspergillus meningitis cases and reported that more than 50% were in individuals without any predisposing factor or known immunosuppression (Antinori et al., 2013). Also, Zhu et al. (2014) have reported that there was no decline in immune function despit brain-occupying lesions caused by Aspergillus infection.

The clinical presentation of patients with CNS aspergillosis is variable and nonspecific (Pettit et al., 2012). Patients typically present with fever, headaches, mental status changes, or seizures. Some patients may show focal neurologic abnormality such as hemiplegia and/or cranial nerve palsy or vision changes (Kourkoumpetis et al., 2012). Usually, an acute course characterized by rapid deterioration of the clinical picture usually ending with death was observed among immunocompromised hosts and in patients with direct inoculation of the fungus into the cerebrospinal fluid or the subarachnoid space. By contrast, a sub-acute or chronic form of meningitis was most frequent among immunocompetent patients, intravenous drug abusers, diabetic patients, and patients who had undergone neurosurgery (Antinori et al., 2013).

CSF examination is often not very helpful in the diagnosis of CNS aspergillosis, since confirmed cases might show negative CSF culture (Kourkoumpetis et al., 2012; Antinori et al., 2013). Isolation of *Aspergillus* from the cerebrospinal fluid is difficult and often requires repeated testing of large volume samples. Other CSF examination findings, such as cell count and protein level, are also nonspecific (Kourkoumpetis et al., 2012).

In our case routine culture plates were negative and failed to isolate *Aspergillus*. The isolation of *Aspergillus* only from CSF put into blood culture bottles raised suspicion that it could be a contaminant. Together the clinical course, CT scan of head showing findings suggesting fungal sinusitis with “bilateral sinupolyposis” and CSF studies (high protein, low glucose, pleocytosis, negative gram stain) with no response to antibiotics, raised the possibility of fungal meningitis. Also, there was a potential source of exposure associated with sinus surgery. The acute course of fungal meningitis with rapid deterioration in immunocompetent patients is observed if direct inoculation of the fungus into the cerebrospinal fluid or the subarachnoid space occurs (Antinori et al., 2013). This prompted us to assume that the patient had significant sinus-associated fungal disease before surgery and that breach in mucosa or direct inoculation of the fungus into the cerebrospinal fluid or the subarachnoid space had occurred during intranasal surgery. The rapidly fatal course of meningitis is consistent with *A. terreus* infection which has a high rate of mortality due to its natural resistance to amphotericin B.

Allergic FRS is a common disorder in patients with sinonasal polyposis. Due to its recurrent and intractable nature, a high degree of clinical suspicion for the presence of FRS in nasal polyposis should be considered (Jain et al., 2013). Although we have no data on histopathology or culture of tissue excised during surgery, the patient history of nasal polyposis together with CT finding suggests the presence of allergic fungal sinusitis. This condition is frequently treated by removing the allergic mucin through surgical procedure. Breach in the mucosal barrier during surgery can result in progression from non-invasive disease to invasive form.

Although *A. terreus* is an unusual cause of CNS invasion (Schwartz et al., 2007) and occurs mostly in immunocompromised patients (Baddley et al., 2003), our case was similar to a case reported by Akhaddar et al. (2008) of invasive *A. terreus* sphenoidal sinusitis with intraorbital and intracranial extension in an immunocompetent patient.

Antinori et al. (2013) has previously suggested that repeated culture of large volumes of CSF (minimum 5 ml) or serial lumbar punctures should be done when mycosis is suspected. In this case standard culturing practice for CSF was negative (incubation period 48 h), while culturing in blood culture bottles was positive after 3 days of incubation, meaning that a prolonged incubation period was needed for the fungus to grow. Cetin et al. (2007) and Udayan and Dias (2014) showed significant increase in isolation rate for BACTEC culture over conventional culture methods from normally sterile body fluids especially from cerebrospinal and synovial fluid specimens. We suggest that culturing CSF in blood culture bottles for more than 48 h should be done if conventional culture is negative and fungal meningitis is suspected.

The detection of aspergillus galactomannan in serum samples by means of an enzyme immunoassay has been validated for the diagnosis of invasive aspergillosis. This assay has also shown promising results with cerebrospinal fluid specimens for the early diagnosis of CNS aspergillosis, although the threshold value for the diagnosis has not been determined (Klont et al., 2004).

The widespread availability of neuroimaging (CT and MRI) leading to earlier radiological diagnosis in these patients, and thus earlier referrals to neurosurgical centers could also be a contributing factor to the rising incidence of reports of Aspergillus CNS infection. Although radiographic imaging may be useful for identifying focal lesions or secondary complications, aspergillus meningitis is usually characterized by an absence of parenchymal lesions (Kourkoumpetis et al., 2012).

Aspergillosis in the CNS carries a poor prognosis, despite the availability of antifungal agents with good activity against aspergillus species and penetration into the CNS (Schwartz et al., 2007). The overall case fatality rate was 72.1% in one report (Kourkoumpetis et al., 2012). The case-fatality of patients with CNS aspergillosis is the highest of all forms of aspergillosis; with mortality as high as 100%. Mortality in patients treated with voriconazole is still unacceptably high, and more effective treatments are needed (Schwartz et al., 2007).

For patients who are intolerant to voriconazole due to hepatotoxicity or other severe reactions, a lipid formulation of amphotericin B (e.g., AmBisome or Abelcet) is a reasonable alternative. The duration of antifungal therapy is dependent upon the location of the infection, the patient's underlying disease, the need for further immunosuppression, and the response to therapy. Antifungal therapy is generally continued until all signs and symptoms of the infection have resolved (Marr et al., 2015).

## Conclusion

The rapid presentation after sinus surgery together with an acute course of meningitis that culmoinated in rapid deterioration and death in this immunocompetent patient was likely caused by *A. terreus* meningitis. It seems likely that undiagnosed fungal sinusitis was complicated by direct inoculation of the fungus into the cerebrospinal fluid during sinus surgery. The rare clinical occurrence of CNS aspergillosis necessitates presentation of case series and case reports so that clinicians can assess the variable presentations of this severe infection. Also, inoculating BACTEC bottles as a means of cultureing CSF fungi needs to be looked into further.

## Funding

This work is partially funded by King Abdul-Aziz City for Science and Technology, Riyadh, Saudi Arabia grant number 12-BIO2295-10.

### Conflict of interest statement

The authors declare that the research was conducted in the absence of any commercial or financial relationships that could be construed as a potential conflict of interest.
